# The brain as an agentic system: how the brain is articulated in the field of neuroenhancement

**DOI:** 10.1111/1467-9566.12810

**Published:** 2018-08-28

**Authors:** Jonna Brenninkmeijer

**Affiliations:** ^1^ Faculty of Science Institute for Science, Innovation & Society Radboud University ‎Nijmegen The Netherlands; ^2^ Faculty of Behavioural and Social Sciences Theory & History of Psychology University of Groningen Groningen The Netherlands

**Keywords:** discourse, media, social representations, science and technology studies, metaphor analysis

## Abstract

This article analyses the material of a European Project on Responsible Research and Innovation in Neuroenhancement (NERRI) to explore how the brain is articulated in this field. Since brains are closely connected to ideas of self, responsibility, free will and being human, and since brain metaphors have important effects on research practices and perspectives, it also matters how people talk about and use the brain. In the NERRI project, the brain is articulated as an agent interacting with or substituting the self; as a system that can, cannot or should not be analysed; and as the part of oneself that can potentially change human nature in positive and negative ways. Since most of the material analysed was produced by neuroscientists or other neuroenhancement experts, this article emphasises the responsibility of the experts in this process. By showing what brain images are disseminated within the field of neuroenhancement, and analysing how this depiction is related to ideas of self or being human, this article does not only intend to contribute to a more empirically based and societally relevant neuroenhancement debate, but also to a more realistic and societally relevant idea of the brain.

## Introduction

Some years ago, I worked as a researcher for two different neuro‐projects simultaneously. In the first half of the week, I analysed transcripts and reports of interviews and debates on the topic of neuroenhancement (the idea that we can use the brain to enhance people's performances), and in the second half of the week, I interviewed neuromarketers and visited conferences on neuromarketing (the idea that we can use the brain to make people buy things). Soon I noticed that both neuro‐fields actually talked about a very different kind of brain. Among neuromarketers and their public the brain was basically articulated as an entity that could be manipulated because of its static, and (evolutionary) determined character (e.g. Morin 2011), while the researchers in the field of neuroenhancement mainly accentuated the plasticity and (future) possibilities of the brain, and referred to people's own responsibility in this process. That is, neuromarketers and neuroenhancement experts appeared to articulate not only different kinds of brains, but also different ways of being human.

Since I was involved in the neuroenhancement project as one of the ‘responsible’ researchers while I was only an observer in the field of neuromarketing,^1^ I decided to analyse the material of this European Project on Responsible Research and Innovation in neuroenhancement (NERRI) to find out how the brain is articulated in this field.^2^ Studying the depiction of the neuro‐enhanceable brain gives information on the meaning of the brain in the field of neuroenhancement, and it also provides information on what the term enhancement does with the idea of the brain. The purpose of this is not only to provide insights in how the brain is evolving in our brain focused society, and how this is related to our ideas of being oneself and being human, but also to reflect on the role of academics in this process.

That scientists do not only study phenomena but also (or rather) constitute these, is a familiar insight among sociologists, science and technology scholars and philosophers (e.g. Latour and Woolgar 1979). In addition, that social scientists also create phenomena with their social studies, such as kinds of people (Hacking 2006), social phenomena (Osborne and Rose 1999) or subjectivities (Pickersgill *et al*. 2011) is also frequently discussed. Hence, this article sees the social scientists, neuroscientists, philosophers, etc., collaborating in the NERRI project not as neutral actors providing objective knowledge about an urgent problem, but as ‘agents of subjectification’ (Pickersgill *et al*. 2011: 362) because they make the public more aware of their brain and the (potential) possibilities to intervene in this brain.

## Humans, enhancement and the brain

Neuroenhancement is the field around the idea that we can use technologies^3^ or pharmaceuticals to enhance our brains, and with this our cognitive or other performances. It is a new (brain) focus in a much older discussion on bioenhancement or human enhancement. So far, these enhancement discussions were mainly hold by bioethicists and philosophers (Pickersgill and Hogle 2015). The topic evolved in the 1990s and has resulted in numerous publications and debates. Many of these, however, seem to repeat the same issues over and over again (Brenninkmeijer and Zwart 2016). The so‐called bioconservatives argue that enhancement technologies will dehumanise people. They will make people less authentic, and society more competitive and unequal (Fukuyama 2002, Kass 2007). On the other hand, Post‐ or transhumanists claim that people should be free to decide for themselves if they want to use enhancers, and that this should not be seen as a degradation, but rather as an upgrade of human nature (Bostrom 2005, Harris 2010). Moreover, many of the arguments in the enhancement debates are mainly theoretical or futuristic (what if), or they discuss the relevance of the debate (does it work/do people really use it) (Hall and Lucke 2010, Quednow 2010).

As a result, some scholars argue that the debate should be refocused to a more realistic and societally relevant stage, and for example, analyse the ideas of the public regarding specific neuroenhancement cases (Brenninkmeijer and Zwart 2016, Coveney 2011). This approach seems to meet the empirical research agenda on enhancement as proposed by Pickersgill and Hogle (2015) in which they suggest that the medical humanities and social sciences should contribute to the social discourse around enhancement – for example, by attending to issues that are not commonly discussed yet, or by studying actual practices. Moreover, one of their suggestions is to study what ‘ideas about society, of the body, and of individual hopes and fears are imagined and/or channelled within processes of innovation’ (p. 137). This article analyses the ideas of the brain that are channelled within the (dissemination) processes of neuroenhancement, and with this reflection, I hope to contribute to a more societally relevant and empirically based neuroenhancement debate.

Moreover, my analysis also contributes to the (sociological) discussions on the impact of neuroscience on human beings. According to many scholars, ideas of the brain are related to ideas of self, personhood or subjectivity. In neuroscience and neurophilosophy, the brain is often presented as the seat of the self and the (supposed) causal functioning of our neuronal system implies that concepts such as free will or responsibility are empty concepts (Churchland 2013, Damasio 2012). Also in other disciplines, such as psychiatry or law, human behaviour is increasingly explained with the help of neuroscience. As a result of such developments, the sociologist Nikolas Rose, argued some years ago that we have become neurochemical selves, because personhood is no longer concerned with the mind or the psyche, but with the brain (Rose 2007).^4^ This new knowledge makes that we take ourselves to be different kinds of persons, and can be understood as a shift in human ontology: ‘It entails a new way of seeing, judging, and acting upon human normality and abnormality. It enables us to be governed in new ways. And it enables us to govern ourselves differently’. (Rose 2007: 192). Other scholars described a comparable impact of brain knowledge on the self. The anthropologist of science Joseph Dumit, for example, studied how brain images can alter people's understanding of their own body (Dumit 2004). Fernando Vidal performed a historical study on ‘brainhood’, with which he referred to the ‘quality of being a brain’ (Vidal 2009). Davi Johnson Thornton analysed how the message that ‘you are your brain’ enables the idea of biological self‐constitution (Thornton 2011). Several other scholars used ethnographic research or media analysis to argue that people who are confronted with their neurological constitution do not simply become neurological subjects but use a heterogeneous language of psychological and physiological statements (Bröer and Heerings 2013, Choudhury *et al*. 2012, O'Connor and Joffe 2013, Pickersgill *et al*. 2011, Singh 2013). Additionally, Rose and Abi‐Rached (2013) extensively describe this development of ‘personhood in a neurobiological age’, and they conclude that personhood has not become brainhood, but that the idea of neuroplasticity requires that people take care of their brains (see also Brenninkmeijer 2010).

That is to say, the impact of brain knowledge on ideas of being human is discussed in many forms. The idea that people are their brains, and that the brain is a causal machine, seems to imply that concepts such as self, free will or responsibility have to be reconsidered. The idea that the brain is plastic and changeable, on the other hand, is often discussed in a neoliberal context in which brain plasticity becomes a(n) (individual) responsibility (Broer and Pickersgill 2015, Malabou 2009, Pitts‐Taylor 2010, Rose and Abi‐Rached 2013). Other empirical studies especially relativise the importance of the brain, or show its impact on subjectivity.

Hence, it was interesting to find that these different characteristics of the brain as related to the self also emerged when studying what ideas of the brain were channelled in the field of neuroenhancement. Moreover, related to the ideas of the brain and its influence on the self, was the theme of the brain as a system: too complex to understand according to some, analysable according to others, and something not to disturb according to again others. That is, this metaphor of a system seems to surpass the well‐known metaphor of the brain as a computer since it is more flexible and less explicative. As Borck (2012) convincingly describes in his chapter on models and metaphors in brain research, brain metaphors are significant because they shape research perspectives and the accompanying neurocultures (see also Williams *et al*. 2012). The combination of these themes resulted in a third theme which was phrased as the natural and artificial brain. This theme about the impact of brain optimisation on human nature and human evolution appeared to be an inheritance of philosophical debates on bioconservatism and transhumanism (e.g. Bostrom 2005).

That is, this article analyses what kind of brain discourse is articulated in an extensive European project on neuroenhancement. Herewith, it aims to contribute to (sociological) discussions on the impact of neuroscience on human beings (e.g. Rose and Abi‐Rached 2013). Since brains are closely connected to ideas of self, responsibility, free will and being human (e.g. Churchland 2013), and since metaphors have important effects on research practices and perspectives (Borck 2012), it also matters how people talk about and use the brain. Moreover, since most of the quotes analysed for this article were produced by neuroscientists or other neuroenhancement experts, this article emphasises the responsibility of the academics in this process. By showing what brain metaphors and promises are disseminated in the neuroenhancement debate this article does not only intend to contribute to a more societally relevant and empirically based neuroenhancement debate (Brenninkmeijer and Zwart 2016, Pickersgill and Hogle 2015), but also to a more realistic and societally relevant idea of the brain.

## Material and methods

The NERRI projected ran from 2013 to 2016 and was funded by the European Commission. According to the Description of Work, NERRI:

aims to contribute to the introduction of Responsible Research and Innovation (RRI) in Neuroenhancement (NE) in the European Research Area and to the shaping of a normative framework underpinning the governance of NE technologies. These will be achieved through mobilization and mutual learning (MML) activities engaging scientists, policy‐makers, industry, civil society groups, patients and the wider public. (NERRI 2013)

The project was executed by a consortium of 18 partner institutions (research, science communication and patient advocacy institutions) from 11 European countries.^5^ Each country collected relevant national literature on neuroenhancement and conducted semi‐structured interviews with stakeholders about their opinions on neuroenhancement. These stakeholders could be researchers or producers of neuro‐technologies, policymakers or ethicists, or (potential) users of neuroenhancement technologies such as patients or students. Summaries of these – over 120 – interviews were collected and analysed and subsumed in a report (NR‐I).^6^

To stimulate ‘Societal Dialogue’, each partner institution organised several mobilisation and mutual learning exercises (MLE's). An MLE ‘aims to bring together various groups of stakeholders (researchers, users, intermediaries, professionals, students, media, broader publics) to facilitate a mutual learning process through mutual exposure of views and experiences, expectations and concerns’ (NERRI 2015). These MLE's were seen as the core of the project and they varied extensively in size (from 7 to 130 participants, and some larger – up to 1200 people – events), duration (mostly less than 1 day, but sometimes a couple of days or even weeks), form (focus groups, science café's, debates, student courses, workshops, symposia, think tanks), means (expert lectures, card games, theatre, cinema) and spoken languages. English reports of these – 64 in total – events were presented in the form of newsletter articles on the website, and analyses and summaries were collected with an online form.

The international and interactive character and the extensiveness of the project make NERRI an interesting and relevant case to study the field of neuroenhancement. I used the written material available in Dutch and English to analyse how the word brain is used in the NERRI reports. This material included the summaries of the interviews, the summaries of the MLE's, the newsletter articles, some notes of NERRI meetings, and the Dutch interviews and transcripts of the MLE's (see Appendix). The material I used (summaries, newsletters, transcripts) was not produced by, nor on authority of, me, since my role was limited to the analysis of the MLE's and other material produced in the project. I was part of NERRI for the Radboud University of Nijmegen (Netherlands), which was responsible for the organisation and analysis of the MLE's. I attended most of the Dutch MLE's, and I had access to the transcripts/recordings of the Dutch MLE's and interviews. I also had a role in the production of a progress and final report on the results of the MLE's (NERRI 2014, 2015).

To analyse this diverse material – consisting of summaries, news reports and full transcripts of interviews or public events – I used a thematic content analysis (e.g. Green and Thorogood 2014). I collected all sentences with the word ‘brain’, added some sentences that were indirectly related to the brain (e.g. by referring to its chemicals or processes), and excluded those phrases that were less meaningful – for example, because the word ‘brain’ was used in a reference, or as part of a technology (deep brain stimulation). Next, I listed all unique brain phrases and after reading and coding (and rereading and recoding), these could be categorised in three themes. To give an indication of the numbers: I collected about 1000 brain quotes (350 pages) which resulted in about 150 unique and relevant quotes. These were divided into three categories of about 40–50 quotes per category, and some remaining quotes. Many quotes expressed the relation between the person (mind, self, sometimes body) and the brain. Other quotes concerned the ‘knowability’ of the brain. A third category of quotes concerned the impact of brain enhancement on human nature.^7^

These themes were recognised inductively and the interviews, events, etc. were also not organised to find information regarding the brain. Moreover, the diversity of the material makes it assumable that not all themes are equally spread among the material. It is possible, for example, that a summary might contain less metaphors, or sentences on person‐brain relations, than a newsfeed, or a transcript. Perhaps a public event evokes more popular metaphors than a personal interview – for example, because they both aim at a different audience. It is also conceivable that in some countries or events the topic human nature is more discussed than in others, or that some people (e.g. neuroscientists) make more brain‐related remarks than others (e.g. philosophers). However, due to the enormous amount of material, and also the overlap (MLE's were summarised, sometimes transcribed and sometimes discussed in newsfeeds), it was not analysed which themes occurred more in which sort of material, event etc.

The NERRI project intended to stimulate a mutual debate among (potential) users, producers, intermediaries and the general public about the topic of neuroenhancement (NERRI 2013). However, the material produced under the name of NERRI mainly derived from NERRI partners (e.g. writing newsletters or reports), and also the transcripts of the interviews and events mainly gave a voice to people who were somehow familiar with the field of neuroenhancement. That is, the NERRI quotes are not general in the sense that they could have been expressed by anyone, but the wide range of countries, events and actors involved, combined with the aim of the project to evoke mutual learning events and to disseminate this knowledge among the general public, indicates that these quotes are at least released within a wide public.

## Brains and selves

One of the themes that was identified by reading through the quotes collected, concerned the brain as being (part of) the mind or self. Analysing these quotes gave insight in to how the NERRI people (and perhaps other people too) express the relation between the brain, the body and the mind or self. In contrast to traditional philosophical debates about dualistic and monistic existences, these quotes revealed a different sort of brain idiom. Instead of meandering between being a brain (monism) or having a brain (dualism), many people seem to have obtained a position transcending these categories.

Many experts (NERRI partners, or experts invited for an interview) emphasised the situation that we are more than our brains. Phrases like: ‘the whole person, not just [on] the brain, mood or body’ (MLER), ‘It is about something much broader than brains’ (I‐1), ‘it is about our brain, not our body’ (NM), ‘our brains and bodies’ (NR‐R) or ‘Not just the body but the mind’ (NN) make clear that according to many people there is more than just the brain. In these quotations, a difference emerges not only between mind and body (as we were used to), but between minds (or moods), bodies and brains. That is, following the meaning of these quotes, people have brains *and* bodies *and* minds. Even in antidualistic explanations this distinction between body and brain comes to the fore. One of the Dutch interviewees, a neuroscientist, for example says: ‘We are not dualists anymore – the idea that our body is something different than who we are. Body and mind are not separate. Both bodily changes and neurological changes change yourself’ (I‐2). This referring to *both* bodily and neurological processes still reveals a dichotomy.

Such expressions about people having brains, bodies and minds support a neo‐Cartesian argument, as, for example, explained by Hacking (2007). According to Hacking, neuroscientists simply added a brain to a dualistic idiom, by which they made a triad of mind, body and brain. This mind‐body‐brain creature is not unique for neuroenhancement experts, but manifests itself also in other brain texts and expressions (Brenninkmeijer 2016). Although this image perhaps evokes a somewhat restricted understanding of human beings – since people often make use of many more entities that cannot simply be defined to a mind‐body‐brain idiom (Brenninkmeijer 2016) – it can be recognised in the NERRI brain quotes.

Moreover, NERRI people do not only express humans as persons with brains, bodies and minds: they also show that there can be interaction among these entities. This interaction occurs in two ways. The most common variant is a caring relationship with the brain, and this especially becomes manifest when people discuss brain enhancement technologies such as neurofeedback or transcranial Direct Current Stimulation. To explain such brain technologies, experts and other people involved in a World Café^8^ talk about the possibilities to, for example, ‘train’ their brain, ‘fool’ their brain, ‘slow down’ their brain or ‘decide for’ their brain (MLE38). Also with regard to ‘more natural’ experienced methods like coffee, sleep, r healthy eating, participants wonder if these cannot help them to ‘achieve these “right” dopamine levels’, according to one of the NERRI partners (MLER).

This caring for the brain is not simply a one‐way action. The brain is also expected to return the favour – for example, in the form of more creativity, intelligence or concentration. Still, it could be argued that this mind‐body‐brain idiom is not much more elucidating than monistic statements that ‘we are our brain’ or dualistic statements that our material body produces an immaterial spirit. ‘If we take care of our brain, our brain increases our performance’ sounds as simple as ‘If we drink much water, our kidneys will produce more urine’. However, while kidneys are relatively predictable servants, brains appear to be more agentic actors (or actants).

Agentic brains represent another subcategory that can clearly be identified in the NERRI expressions. This acting is often formulated with phrases like: ‘people with …. brains’. Examples given by NERRI partners or experts involved in NERRI debates are: ‘A person with a “criminal” brain’ (W, MLE3), ‘People with dopamine deficiency’ (MLE1) or ‘People with less beta‐activity [are more vulnerable to complaints of burn‐out]’ (MLE38). Expressions from experts with a comparable meaning are among others: ‘it [only] works for some people who are below a certain level of neurotransmitters’ (I‐3), or ‘everything we observe, experience, process, keep, think about, say, sing, our motor system: everything is in principle steered by the brain’. (I‐4)

In some cases, this agentic brain can take over human actions or even exclude the self at all. Neuroscientists and other neuro‐experts (practitioner; student) used phrases like: ‘In the case of acute stress, the brain takes the prefrontal cortex offline’ (MLE1), ‘Dopamine (…) makes sure you can do better’ (I‐2), ‘What brains can do with us’ (MLE38), ‘the brain is smart, it can recover’ (MLE39) or ‘Our brain, just like other systems, is something that strives for homeostasis. Self‐preservation’ (MLE1). This indicates that brains can be dominant actors, overruling the need of a person or self at all.

Moreover, in some phrases, brains even acquire human qualities, or start to interact with the owner of the brain. One producer of neuroenhancement technologies says, for example, in an interview: ‘all our little neurons are correlation seekers’(I‐5) and ‘there will be neurons that recognise this very fast’(I‐5). In a news report on the NERRI website, a neuroscientist talks about an ‘Immoral molecule’ (NN). Such phrases attribute human capacities directly to the brain. Other neuroenhancement experts made phrases like: ‘when the brain doesn't like it (…), it can give you a headache’ (MLE38) or ‘It changes the working of your brain so much (that) it affects how you lead your life’ (I‐1). This reveals an interaction between a human actor (you) and a human brain.

This interaction between the self (you) and the brain can be so prominent that actually two autonomous actors emerge – both performing not only in a caring but also in a competitive relationship. This becomes salient in this quote of a neurofeedback therapist involved in one of the workshops: ‘If someone has something with his brain, but works very hard, perhaps he compensates and you don't notice anything. While someone with an optimal brain, who cuts corners, might be less efficient’ (MLE38). That is, in this quotation it is impossible to maintain a monistic argument since the brain and the self can compensate each other's actions.

The idea of neuroenhancement clearly emphasises the notion of brain plasticity (we can improve our brains), but this view is regularly combined with more deterministic understandings (we are our brains). Apparently, the self and the brain can be separate *and* the same. The brain is not a static actor, but is extremely flexible and active: it can be you or producing you, is both subject and object, figures as master or servant, has human qualities or disrupts these, produces free will or eliminates it. Moreover, instead of being a brain or having a brain, people seem to develop an agentic brain capable of interacting with or substituting the self.

## Brain systems and metaphors

Above, I gave an account of the interactions between the brain and the self, as they appeared in my analysis of the NERRI quotations. However, being, having or interacting with the brain does not provide much information yet about people's conceptions or understandings of the brain. In this section, I analyse those brain quotations that referred to the (desired or expected) ‘knowability’ of the brain.

Comparing all brain‐related quotations from the NERRI project, a general picture of the brain as a system emerged. Although a system is perhaps not considered as a clear metaphor, this image is most likely influenced by the well‐known metaphor of the brain as a computer (Borck 2012, Rodriguez 2006) and probably also by biological, evolutionary or cybernetic vocabularies that portray the brain as a physiological, adaptive system (Borck 2012, Pickering 2010). Thinking about the brain as a system suggests that we see the brain as a mechanism; as something with input and output. A system is related to the idea of control – although it is not always specified how this control takes place. It is about action and reaction. Since a system is composed of structures and components, in theory we can analyse or understand the system.

In the NERRI reports, this idea of the brain as an analysable system emerges in quotations of NERRI partners and neuroscientists:

‘the processes in the brain would be modelled with high speed computing systems, and subsequently a decoding of the functions of the brain would be possible’ (NN)

‘the brain is a highly complex non‐linear system’ (NN)

‘Our brains are like a jig‐saw consisting of many small fragments that work with each other’ (NN)

‘Our brain, just like other systems, is something that strives for homeostasis’ (MLE1)

‘the mechanisms by which the working of the brain gives rise to the mind’ (MLE12)

‘the brain takes the prefrontal cortex offline’ (MLE1)

‘the technical aspects of the (…) working of the brain’ (MLER)

In these explanations, terms like processes, modelling, decoding, nonlinear, jigsaw, fragments or mechanisms reveal a technical interpretation of the brain. The brain is understood as an entity that can be known and probably modified. However, this image of a knowable brain also frightens people, and sometimes goes along with ideas of brain technologies as a sort of lie detectors. One participant of an MLE, for example, fantasises about job applications with EEG: ‘when you have too many high voltages, we don't give you the job because you are susceptible for getting a burn‐out’ (MLE38). A philosopher attending the same workshop suggests: ‘I think your brain structure will be different when you read a lot, or when you play a lot of games or use tablets. I assume. You might be able to see this at the dendrites of the brain’ (MLE38).

The idea of the brain as an analysable system brings along two possible future scenarios: if we understand the brain we might be able to improve the person, but we might also be able to know or control the person. This interrelation of the brain and the person is typical for this specific organ, and radically differs from any other organ in our body. We are happy to know the working of the kidneys, the heart, the lungs, etc., but understanding the mechanisms of the brain also frightens. As a result, two other tendencies regarding the brain as a system can be observed: the brain is often presented as an unanalysable system, a black box. Furthermore, the brain is sometimes described as a holy system: a sanctum we should leave untouched.

The image of the brain as an unanalysable system is expressed by emphasising the ‘complexity’ of the brain, for example, by arguing that even the ‘most prominent researchers are highly sceptical about the prospects of enhancing the healthy human brain’, or that we ‘hardly know what is inside the brain’ (NERRI partner in MLE12). Moreover, professional users and producers of brain technologies who have high hopes of future possibilities for neuroenhancement also often put their ideas into perspective with remarks like: ‘we are still over 100 years away from being able to record from all the neurons in the cortex’ (NN), or ‘The language of the brain is not known yet’ (I‐4).

Sometimes expressed as a result of this complexity, other times as a result of its close relation with personhood, the brain is also seen as an object we should be very careful with. In the NERRI report that summarises the interviews, one of the partners refers to ideas of the brain as a ‘special or almost “holy” organ, and not something to be messed with’ (NR‐I). One philosopher and participant of a Dutch workshop calls this ‘brain exceptionalism’: ‘if it is about the brain, we have to be very careful, and take more precautions’ (MLE38). This idea can be retrieved in several expressions of other people attending this World Café^9^ that mainly involved neuroenhancement experts (students, researchers and practitioners). For example, one participant suggests: ‘In the brain … I have the feeling that it is deeper. It is more irreparable or more disastrous if it goes wrong’ (MLE38). Someone else clarifies: ‘The brain is something special. (…) There is something with identity. It is so much more exciting than a stomach, intestine, or liver. Precisely because it is so important, you make exceptions for it’ (MLE38). This exceptionalism is also phrased in sentences like: ‘But in this case we are really talking about the brain’(MLE38), ‘The brain is (…) very vulnerable and a sensitive organ’ (MLE38), ‘we should be very careful with the brain’ (MLE38) or ‘Do you think we should create a sanctum for the brain?’ (MLE38).

These portrayals of the brain as an analysable system, a black box, or a sanctum are sometimes also reflected in the metaphors people use to describe the brain or its actions. Some neuroscientists, for example, used phrases like ‘pump external signals in the brain’ (I‐5), ‘Dopamine stokes a fire (in the reptilian brain)’ (MLE1), ‘the brain takes the prefrontal cortex offline’ (MLE1), ‘warm up (…) the parietal cortex’ (I‐5). Such expressions derive from metaphors of the brain as an engine, computer or other machinery, and this represents a technical and hence knowable brain that can be tinkered with. A brain that can be ‘impoverished’ (MLE38), ‘diseased’ (NR‐B) or ‘affected’ (NN), or should be ‘protected’ (NN) or ‘repaired’ (NN) on the other hand, indicates a source that should be taken care of since it otherwise could break down or become ill. What is more, these images and metaphors appear to be related to a philosophical discussion about the human as a natural or artificial being. The technical brain seems to represent the artificial human – always seeking for an upgrade, while the brain as a black box or a sanctum represents a respect for the human brain as a balanced and natural entity – something not to disturb.

## Natural and artificial brain

The distinction between a technical or artificial brain (a brain that is considered as having potential beyond normality) and a natural brain (a brain that should not be disturbed because it needs a natural balance to produce authentic human capacities) actually intermingles three philosophical or ethical issues. One on human authenticity that discusses if using artificial technologies can evoke authentic emotions (e.g. Bolt 2007, Kraemer 2011), one on human nature that questions if neuroenhancement is part of a natural process or something that exceeds humanity and makes us ‘post’‐humans (Bostrom and Sandberg 2009, Clark 2003) and one on human evolution that questions if we should protect human beings from becoming post‐ or transhuman (e.g. Fukuyama 2002, Kass 2007) or that we should stimulate this transition (Bostrom 2005, Harris 2010).

In the NERRI project, the discussion about the brain as a natural or artificial object is often formulated as a debate about neuroenhancement being part of, or as surpassing, humanity or human evolution. In more implicit terms, this discussion focuses on neuroenhancement as belonging to the normal human being (as functioning within the limits of normal human behaviour; as a normal thing to do), or as something that will exceed normality. Classifying NERRI quotes regarding this natural/artificial theme, however, does not show a clear division in being pro or contra neuroenhancement. The argument that neuroenhancement is a normal or natural human habit is often used as an argument to speak in favour of neuroenhancement, but it can also exemplify a warning that we should not exaggerate the possibilities of enhancement and be alert to the safety problems. The argument that we can become super‐humans, on the other hand, is often (but not always) expressed to unfold a dystopian scenario.

Quotes from NERRI news reports referring to neuroenhancement as a typically human thing to do or as belonging to a normal evolutionary process are often variations of the argument: ‘Humanity has been always searching for means to enhance further brain performance’. (NN) or ‘Enhancing the human brain is as old as humanity itself’ (NN). Other quotations emphasise the unicity of human qualities: ‘The ability to improve ourselves is unique for human beings’ (Neuroscientist in MLE1) or make a strong connection between enhancement and civilisation: ‘Human civilization is basically a series of experiments in enhancement’ (NN). Moreover, in some expressions, neuroenhancement as part of this evolutionary process is also questioned. Phrases from NERRI news reports illustrate this uncertainty: ‘Will we need it [neuroenhancement] for the survival of our species?’ (NN), ‘Aren't humans always “artificial”?’ (NN), ‘can we make significant changes to what nature gave us and still call ourselves humans?’ (NN). Also, students who follow a neuroenhancement course related to MLE1 wonder in an essay: ‘How authentic can grandmother be if her Alzheimer's is cured, but half of her brain is artificial?’(MLE1a).

The majority of quotes in this direction, however, represent an opposite position. In most occasions, neuroenhancement is not considered as a normal human process but as something surpassing humanity or human evolution. The NERRI website, for example, makes claims like: ‘We are entering a new chapter in the human history, we can become transhumans’ (NN) or ‘a normal human is just not able to see like this’ (NN). This represents a point of view which is occasionally brought up as very positive. Neuroenhancement, for example, ‘may allow’ humans to perceive ‘beyond the scope of our biological organs’ (NN), and makes persons ‘able to realize achievements’ (MLE1a) or ‘complement’ (NERRI partner in MLE12) their biological brains. The artificiality argument is sometimes also formulated as a question, (‘do we want a super brain?’ [MLER]) – but it is most often related to concerns and objections towards neuroenhancement. Fears that people can become ‘outdated’, dangerous scenarios about ‘brains with electronic components’, ‘hyperintelligent super‐brains’ or ‘the robotization of humans’, represent such dystopian science fiction scenarios as phrased by NERRI partners (W, MLER). On the other hand, fears about changing ‘the natural cycle of life’, the ‘natural balanced state of the human brain’, the ‘natural balance’ or the ‘natural human evolution’ (MLER) – actually make the same argument as the dystopian science fiction scenarios, but emphasises an opposite characteristic of the brain (i.e. natural instead of artificial).

The distinction between natural and artificial brains does not follow a clear line between proponents and opponents of neuroenhancement. In contrast to what might be expected from philosophical discussions on authenticity, human nature or human evolution, the distinction between the human (or brain) as a natural being that should be authentic and not something to (artificially) tinker with, or the brain as the point of access to a new evolutionary process; a plea for transhumanism – is not very clear. The idea that neuroenhancement is a normal process in human evolution is often used as a pro‐enhancement argument, while the idea that neuroenhancement surpasses normality or human evolution merely evokes negative associations towards neuroenhancement. However, what is quite specific for this theme of natural and artificial brains is that the idea of brain optimisation seems to be related to our understanding of being human. Improving brain capacities is considered as elevating us above a ‘natural’, ‘normal’ human level. The brain is seen as the part of ourselves that grants us our humanity and hence, tinkering with the brain is also seen as tinkering with humanity or human evolution.

## Worrying about the brain

In the NERRI project, the brain is articulated as an agent interacting with or substituting the self. It is seen as a system that can, cannot or should not be analysed. In addition, the idea of neuroenhancement turns the brain into an object that can potentially change human nature in positive and negative ways. However, this analysis leads to some further questions. One might wonder, for example, how general these tendencies of the brain are, and what these conclusions actually entail.

Most of the quotes used in this article stem from people who can be seen as neuroenhancement experts. Many of them are from NERRI‐partners; writing newsletters for the website, giving summaries of MLE's, or participating in their own debates. Others are from neuroscientists, being interviewed, giving lectures, or participating in focus groups. Furthermore, quotes are from students following a course on neuroenhancement, or from producers, practitioners or users of neuroenhancement technologies. Taking together all these quotes from all these experts figuring in many different events, situations and texts, gives a rough exposition of brain related expressions that generally do not represent the current state of affairs in neuroscience or philosophy. On the contrary, this article makes clear that scientific or philosophical categories or dichotomies do not always persist when they are released in the general public. Classic monistic and dualistic idioms are intermingled, the brain is explained with fantastic metaphors, and ethical arguments on authenticity and artificiality do not always follow the expected lines. As a result, such brain expressions could easily be interpreted as small jokes or imprecisions, as slips of the tongue without any implication. However, I would like to argue that these explanations, metaphors and concerns are significant, and perhaps especially because they derive from the voices of neuroenhancement experts. As indicated among others by Osborne and Rose (1999), (social) scientists can also create phenomena, and although neuroenhancement experts will have no intention to disseminate an agentic version of the brain, their ‘neuroenhancement gaze’ might help constituting a specific way of thinking about the brain (Pickersgill *et al*. 2011).^10^

New knowledge, metaphors or ideas about the brain do not change the brain as a substance. They might, however, change the role the brain performs in our lives and society. The brain as an agentic system capable of threatening or saving human nature is something completely different than the brain as a computer or as a static (or declining) organ. These different portrayals of the brain interact with different actors (ethicists, neuroscientists instead of, for example, clinical neurologists), might influence policy (the aim of NERRI), evokes new knowledge (e.g. on free will and responsibility) and emotions (e.g. fears and hopes). That is, although the brain as an agentic system that makes us human has the same biochemical constitution as the brain we study for medical reasons or biological knowledge it clearly interferes with other actors, policies, knowledge, emotions, responsibilities, etc. Not only the ‘idea’ of the brain differs in these contexts: but what the brain is and what the brain does (See e.g. Mol and Law 2004). Hence, the aim of the NERRI project to create awareness and responsibility regarding neuroenhancement technologies through engaging scientists, policymakers, industry, civil society groups, patients and the wider public – makes it assumable that this eventually also affects the role of the brain in our lives and society.

## Conclusion

One of the aims of this article was to contribute to insights in how the impact of neuroscience influences people's ideas of self (e.g. Rose and Abi‐Rached 2013). I demonstrated that NERRI people articulated a brain with several characteristics. In their representations, the brain becomes agentic. In some expressions, it becomes an entity that interacts with the body and/or self, and in other sentences it takes over human actions or substitutes the self. This shows that the idea of neuroenhancement clearly emphasises the notion of brain plasticity (we can improve our brains), but that this view is regularly combined with more deterministic understandings (we are our brains). Moreover, the situation that many of the quotes presented were expressed by neuroscientists or other neuroenhancement experts demonstrates that this idea of an agentic brain that can interact with the self and the body, and hence makes people (only) occasionally responsible and free is not a confusion due to a misunderstanding of science – it are academics themselves who articulate the brain in this way.

Another characteristic of the brain was the idea of the brain as a system; that can, cannot, or should not be analysed. This idea of the knowable brain mainly frightens because it is related to the idea of knowable and controllable people. Again, this is not a sort of naïve folk anxiety for neurologists, but is inferred from explanations of neuroenhancement experts. When we look at daily neuroimaging practices, however, the knowable brain is by far not in reach (e.g. Poldrack *et al*. 2018). That is, the brain as a system breaths the hopes and fears of future possibilities, but seems to have less in common with its current position in the laboratory. This is the same with the third characteristic that I recognised in the NERRI quotes: the brain as the part of ourselves that can make the distinction between natural and artificial. This idea, that the brain makes us part of nature and humanity, and hence could also change our humanity is fascinating, but has nothing to do with daily scientific practices or results.

What can be concluded is that the brain as an agentic system that has the potential to influence human nature is a result of intermingled philosophy, metaphorical neuroscience and imaginative ethics. Although this article does not demonstrate that the brain as an agentic system is a reality among the European public, it seems to be a reality among European neuroenhancement experts. Moreover, the more this depiction is spread the more real it will become. What the impact is of a brain that can overrule the self, is potentially controllable, and can change humanity is speculation, but it will probably not make people more creative and autonomous. However, instead of constituting a new piece of imaginative ethics, I hope this article will contribute to some reflection among the researchers working in these neuro‐fields. To make the debate on neuroenhancement more empirically based and societally relevant, I propose that we could start with a more empirically based (and societally relevant) explanation of the brain.

## Notes

1 For more information about the brain in the field of neuromarketing, see Brenninkmeijer, J., Schneider, T and Woolgar, S. (Under Review) ‘Witness and silence in neuromarketing: managing the gap between science and its application’.

2 Unfortunately, the old NERRI website is no longer available but can still be consulted at: https://web.archive.org/web/20160729102025/http://www.nerri.eu:80/eng/home.aspx (Last accessed on February 2018).

3 For example, transcranial Direct Current Stimulation, neurofeedback, Transcranial Magnetic Stimulation, Deep Brain Stimulation.

4 In Rose and Abi‐Rached (2013), this argument was nuanced: ‘[this term'neurochemical self’] was meant to imply, not a wholesale mutation in personhood, but the availability of a neurochemical register within which individuals could describe, judge, and seek to modulate their mental states and ailments’. (p. 272)

5 Ciência Viva – Agência Nacional para a Cultura Científica e Tecnológica, Portugal; London School of Economics and Political Science, United Kingdom; Radboud University Nijmegen, Netherlands; Austrian Academy of Sciences, Austria; Scuola Internazionale Superiore di Studi Avanzati, Italy; Instituto de Biologia Molecular e Celular, Portugal; Experimentarium, Denmark; Tilburg Law School – Tilburg University, Netherlands; Central European University, Hungary; Universitat Stuttgart, Germany; Johannes Gutenberg Universitat Mainz, Germany; Johannes Kepler Universitat, Austria; Universitat Pompeu Fabra, Spain; Toscana Life Sciences, Italy; University of Iceland, Iceland; Genetic Alliance UK, United Kingdom; European Brain Council, Belgium; King's College London, United Kingdom.

6 See Appendix for an explication of these abbreviations.

7 This was at the end of my analysis. I started with many codes that I divided into the categories: Having a brain, interacting with your brain, being your brain; Acting brains; Brain as a black box, brain as an analysable (controllable) system, brain as a relic; The natural brain, the artificial brain; NE as part of humanity/evolution; NE as surpassing humanity/normality; others. This resulted in four themes: Brain beings, Brain images, The natural brain vs. the artificial brain, Metaphors. Also, these were thereupon reclassified under the themes of the current paper: Brains and Selves; Systems and Metaphors; Natural and Artificial brain.

8 In a world café people (experts, students, producers) discuss in small groups, in changing formats, about several issues in neuroenhancement. This is different from a science café where the audience go into discussion with scientists (neuroenhancement experts) in public.

9 See note 14.

10 Pickersgill and colleagues actually make a different argument. They claim that individuals do not uncritically adopt neuroscientific knowledge to their self‐understanding but that they make their own bricolage of knowledge. They also claim that it is partly through the sociological gaze itself that neurologic subjectivity is constituted. I use this last argument to argue that neuroenhancement experts also create new ideas about the brain. This maintains the argument of Pickersgill *et al*. (2011) that this does not necessarily mean that this idea of the brain is also uncritically adopted by the general public.

## Explanation of the material

### Documents produced by NERRI partners

MLER = Full Reports of NERRI Mutual Learning Exercises, produced by NERRI partners and collected with an online questionnaire. In February 2018 to be found at: https://web.archive.org/web/20160810054107/http://nerri.eu/eng/mutual-learning-exercises.aspx

NN = NERRI news, written by NERRI partners on the NERRI website. Collected in March 2016 from: http://www.nerri.eu/eng/news-highlights/nerri-news.aspx. In February 2018 to be found at: https://web.archive.org/web/20160324061457/http://nerri.eu/eng/news-highlights/nerri-news.aspx

NM = NERRI meeting, May 2014 (notes of the author).

NR = NERRI Reports.

I = Interview Report: NERRI D2.3 National and European perspectives of stakeholders. Summaries of all interviews from all countries, written by NERI partners
R = Reconnaissance: D2.2 Neuroenhancement and European Normative Anchor Points
B = D2 5_NERRI_Briefing_Paper

### Interviews (Neuroenhancement experts, not involved in NERRI)

I = Dutch interviews, (10 interviews conducted by Winnie Toonders, transcribed, originally in Dutch). In this article are quoted:

1. Researcher of neuro‐technologies (student)
2. Neuroscientist (Professor)
3. Researcher of neuroenhancement (student)
4. Cognitive neuroscientist (Professor)
5. Investigator of neuro‐technologies (Professor)

### Mutual Learning Exercises (Experts and Public)

MLE1 = Science Café Nijmegen (recorded and transcribed, originally in Dutch; 9 pages).

MLE1a = Course material (90 pages; including essays written by students), related to MLE1. See Toonders *et al*. (2016) for more information on this course and MLE.

MLE3 = DBS Symposium Tilburg (notes, originally in Dutch, 8 pages).

MLE12 = Neuroenhancement: a true unfolding of man. Partly transcribed in November 2014 from https://www.youtube.com/watch?v=Eioy_sIICiU (7 pages).

MLE38 = World Café Enschede EEG (recorded and transcribed, originally in Dutch; 76 pages).

MLE39 = World Café Enschede tDCS (recorded and transcribed, originally in Dutch; 57 pages).

### Summary of the material

**Table 1:**

Code	Description	Produced by:	Pages
MLER	Full Reports of NERRI Mutual Learning Exercises	NERRI partners	235
NN	NERRI news	NERRI Partners	143
NM	NERRI Meeting, notes	NERRI Partners	13
NR (I/R/B)	NERRI Reports: Interview Summaries (I) Reconnaissance (R) and Briefing paper (B)	NERRI Partners	207
I (quotes from 1 to 5 are used)	Interview transcripts of 10 interviews + notes	Neuroenhancement Experts	49
MLE (1, 3, 12, 38, 39)	Mutual Learning Exercises, notes and transcripts	Experts and public	247

## References

[shil12810-bib-0001] Bolt, L.L.E. (2007) True to oneself? Broad and narrow ideas on authenticity in the enhancement debate, Theoretical Medicine and Bioethics, 28, 4, 285–300. 17909988 10.1007/s11017-007-9039-8 PMC2798025

[shil12810-bib-0002] Borck, C. (2012) Toys are us: models and metaphors in the neurosciences In Choudhury S. and Slaby J. (eds) Critical Neuroscience: A Handbook of the Social and Cultural Contexts of Neuroscience, pp. 113–23. London: Blackwell.

[shil12810-bib-0003] Bostrom, N. (2005) In defense of posthuman dignity, Bioethics, 19, 3, 202–14. 16167401 10.1111/j.1467-8519.2005.00437.x

[shil12810-bib-0004] Bostrom, N. and Sandberg, A. (2009) Cognitive enhancement: methods, ethics, regulatory challenges, Science and Engineering Ethics, 15, 3, 311–41. 19543814 10.1007/s11948-009-9142-5

[shil12810-bib-0005] Brenninkmeijer, J. (2010) Taking care of one's brain: how manipulating the brain changes people's selves, History of the Human Sciences, 23, 107–26. 20518157 10.1177/0952695109352824

[shil12810-bib-0006] Brenninkmeijer, J. (2016) Neurotechnologies of the Self: Mind, Brain and Subjectivity. London: Palgrave Macmillan.

[shil12810-bib-0007] Brenninkmeijer, J. and Zwart, H. (2016) From ‘hard’ neuro‐tools to ‘soft’ neuro‐toys? Refocussing the neuroenhancement debate, Neuroethics, 10, 3, 337–48. 28890737 10.1007/s12152-016-9283-6 PMC5569123

[shil12810-bib-0008] Bröer, C. and Heerings, M. (2013) Neurobiology in public and private discourse: the case of adults with ADHD, Sociology of Health and Illness, 35, 49–65. 22738075 10.1111/j.1467-9566.2012.01477.x

[shil12810-bib-0009] Broer, T. and Pickersgill, M. (2015) Targeting brains, producing responsibilities: the use of neuroscience within British social policy, Social Science and Medicine, 132, 54–61. 25792340 10.1016/j.socscimed.2015.03.022

[shil12810-bib-0010] Choudhury, S. , McKinney, K.A. and Merten, M. (2012) Rebelling against the brain: public engagement with the “neurological adolescent”, Social Science and Medicine, 74, 565–73. 22257745 10.1016/j.socscimed.2011.10.029

[shil12810-bib-0011] Churchland, P.S. (2013) Touching a Nerve: The Self as Brain, 1st edn New York: W. W. Norton and Company.

[shil12810-bib-0012] Clark, A. (2003) Natural‐Born Cyborgs:Minds, Technologies, and the Future of Human Intelligence: Minds, Technologies, and the Future of Human Intelligence. USA: Oxford University Press.

[shil12810-bib-0013] Coveney, C.A. (2011) Cognitive enhancement? Exploring modafinil use in social context In Pickersgill M. and Van Keulen I (eds.) Sociological Reflections on the Neurosciences, Vol. 13, pp. 203–28. Bingley: Emerald Group Publishing Limited.

[shil12810-bib-0014] Damasio, A. (2012) Self Comes to Mind: Constructing the Conscious Brain. New York City: Random House Incorporated.

[shil12810-bib-0015] Dumit, J. (2004) Picturing Personhood: Brain Scans and Biomedical Identity. Princeton: Princeton University Press.

[shil12810-bib-0016] Fukuyama, F. (2002) Life, but not as we know it, New Scientist, 174, 2339, 42. 12426701

[shil12810-bib-0017] Green, J. and Thorogood, N. (2014) Qualitative Methods for Health Research, 3rd edn Los Angeles: SAGE.

[shil12810-bib-0018] Hacking, I. (2006) Making up people, London Review of Books, 28, 16, 23–6.

[shil12810-bib-0019] Hacking, I. (2007) Our neo‐Cartesian bodies in parts, Critical Inquiry, 34, 78–105.

[shil12810-bib-0020] Hall, W.D. and Lucke, J.C. (2010) The enhancement use of neuropharmaceuticals: more scepticism and caution needed, Addiction, 105, 12, 2041–3. 21054609 10.1111/j.1360-0443.2010.03211.x

[shil12810-bib-0021] Harris, J. (2010) Enhancing Evolution: The Ethical Case for Making Better People. Princeton: Princeton University Press.

[shil12810-bib-0022] Kass, L.R. (2007) Defending human dignity, Commentary, 124, 5, 53.

[shil12810-bib-0023] Kraemer, F. (2011) Authenticity anyone? The enhancement of emotions via neuro‐psychopharmacology, Neuroethics, 4, 1, 51–64. 21475717 10.1007/s12152-010-9075-3 PMC3053456

[shil12810-bib-0024] Latour, B. and Woolgar, S. (1979) Laboratory Life: The Construction of Scientific Facts. Princeton: Princeton University Press.

[shil12810-bib-0025] Malabou, C. (2009) What Should We Do With Our Brain?. New York City: Fordham University Press.

[shil12810-bib-0026] Mol, A. and Law, J. (2004) Embodied action, enacted bodies: the example of hypoglycaemia, Body and Society, 10, 2–3, 43–62.

[shil12810-bib-0027] Morin, C. (2011) Neuromarketing: the new science of consumer behavior, Society, 48, 2, 131–5.

[shil12810-bib-0028] NERRI (2013) Neuroenhancement: Responsible Research and Innovation. Annex I ‐ Description of Work. 2nd Revised Draft for the Project Officer's consideration, *FP7‐Science‐in‐Society‐2012*, version 8 Nov.

[shil12810-bib-0029] NERRI (2014) D3.3 Progress Report.

[shil12810-bib-0030] NERRI (2015) D 3.5 Final Report NERRI WP3. Available at: https://www.researchgate.net/profile/Hub-Zwart/publication/296703510_NERRI_neuroenhancement_Responsible_Research_and_Innovation_FINAL_Report_WP3/links/56d9979908aee73df6cf593d.pdf (Last accessed on June 2017).

[shil12810-bib-0031] O'Connor, C. and Joffe, H. (2013) How has neuroscience affected lay understandings of personhood? A review of the evidence, Public Understanding of Science, 22, 254–68. 23833053 10.1177/0963662513476812 PMC4107825

[shil12810-bib-0032] Osborne, T. and Rose, N. (1999) Do the social sciences create phenomena? The example of public opinion research, The British Journal of Sociology, 50, 3, 367–96. 15259192 10.1111/j.1468-4446.1999.00367.x

[shil12810-bib-0033] Pickering, A. (2010) The Cybernetic Brain. Sketches of another future: University of Chicago Press.

[shil12810-bib-0034] Pickersgill, M. and Hogle, L. (2015) Enhancement, ethics and society: towards an empirical research agenda for the medical humanities and social sciences, Medical Humanities, 41, 2, 136–42. 26260624 10.1136/medhum-2015-010718 PMC4717454

[shil12810-bib-0035] Pickersgill, M. , Cunningham‐Burley, S. and Martin, P. (2011) Constituting neurologic subjects: neuroscience, subjectivity and the mundane significance of the brain, Subjectivity, 4, 346–65.

[shil12810-bib-0036] Pitts‐Taylor, V. (2010) The plastic brain: neoliberalism and the neuronal self, Health, 14, 6, 635–52. 20974696 10.1177/1363459309360796

[shil12810-bib-0037] Poldrack, R.A. , Monahan, J. , Imrey, P.B. , Reyna, V. , *et al* (2018) Predicting violent behavior: what can neuroscience add?, Trends in Cognitive Sciences, 22, 2, 111–23. 29183655 10.1016/j.tics.2017.11.003 PMC5794654

[shil12810-bib-0038] Quednow, B.B. (2010) Ethics of neuroenhancement: a phantom debate, BioSocieties, 5, 1, 153–6.

[shil12810-bib-0039] Rodriguez, P. (2006) Talking brains: a cognitive semantic analysis of an emerging folk neuropsychology, Public Understanding of Science, 15, 3, 301–30.

[shil12810-bib-0040] Rose, N. (2007) The Politics of Life Itself: Biomedicine, Power, and Subjectivity in the Twenty‐First Century. Princeton: Princeton University Press.

[shil12810-bib-0041] Rose, N. and Abi‐Rached, J.M. (2013) Neuro: The New Brain Sciences and the Management of the Mind. Princeton: Princeton University Press.

[shil12810-bib-0042] Singh, I. (2013) Brain talk: power and negotiation in children's discourse about self, brain and behaviour, Sociology of Health and Illness, 35, 6, 813–27. 23094965 10.1111/j.1467-9566.2012.01531.x PMC3757316

[shil12810-bib-0043] Thornton, D.J. (2011) Brain Culture: Neuroscience and Popular Media. New Brunswick: Rutgers University Press.

[shil12810-bib-0044] Toonders, W. , Verhoeff, R.P. and Zwart, H. (2016) Performing the future, Science and Education, 25, 7–8, 869–95.

[shil12810-bib-0045] Vidal, F. (2009) Brainhood, anthropological figure of modernity, History of the Human Sciences, 22, 1, 5–36. 19860032 10.1177/0952695108099133

[shil12810-bib-0046] Williams, S.J. , Higgs, P. and Katz, S. (2012) Neuroculture, active ageing and the ‘older brain’: problems, promises and prospects, Sociology of Health & Illness, 34, 64–78. 21689114 10.1111/j.1467-9566.2011.01364.x

